# Supplementary material for: B cell receptor dependent enhancement of dengue virus infection
Source: PLoS Pathog. 2024 Oct 31;20(10):e1012683. doi: 10.1371/journal.ppat.1012683 (PMC11556684; doi:10.1371/journal.ppat.1012683)
Supplement: S1 Fig — A) Expression and gating of tmIgG and DC-SIGN in transfected 293T cells B) Representative flow cytometry plots showing the frequency of DENV-1 RVP infected cells within the receptor-negative gate of tmIgG and DC-SIGN in transfected 293T cells 24hrs after RVP exposure C) Quantification of DENV-1 RVP infected cells within the receptor-negative gate of tmIgG and DC-SIGN in transfected 293T cells 24hrs after RVP exposure (PDF) [file ppat.1012683.s001.pdf]

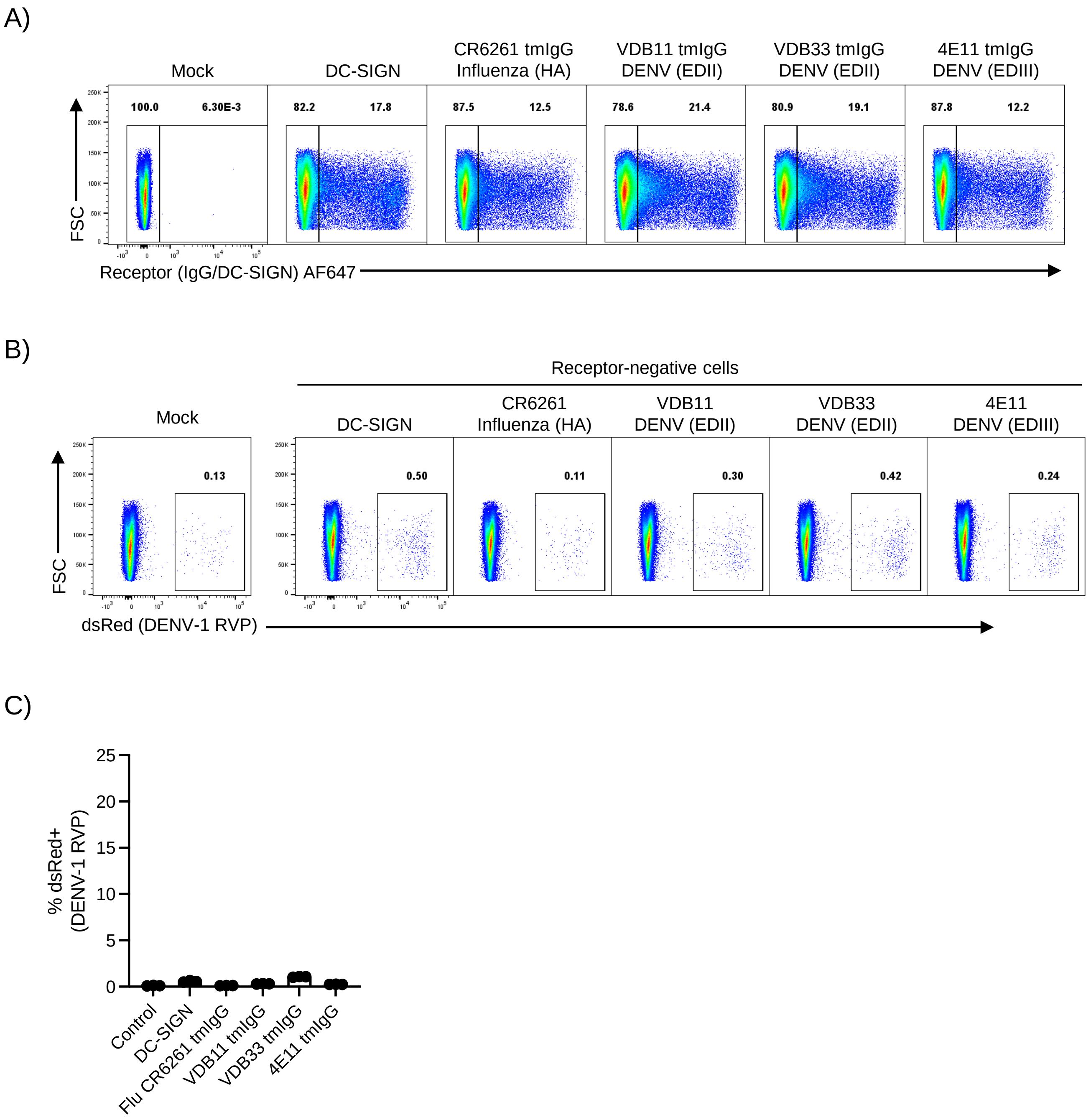

S1 Fig. Gating scheme and DENV-1 RVP infection of tmIgG transfected 293T cells. A) Expression and gating of tmIgG and DC-SIGN in transfected 293T cells **B)** Representative flow cytometry plots showing the frequency of DENV-1 RVP infected cells within the receptor-negative gate of tmIgG and DC-SIGN in transfected 293T cells 24hrs after RVP exposure **C)** Quantification of DENV-1 RVP infected cells within the receptor-negative gate of tmIgG and DC-SIGN in transfected 293T cells 24hrs after RVP exposure
