# Supplementary material for: B cell receptor dependent enhancement of dengue virus infection
Source: PLoS Pathog. 2024 Oct 31;20(10):e1012683. doi: 10.1371/journal.ppat.1012683 (PMC11556684; doi:10.1371/journal.ppat.1012683)
Supplement: S2 Fig — A) Expression and DENV E protein binding activity of VDB33 tmIgM and tmIgA expression constructs. B) Expression and gating of tmIgM and tmIgA expression in transiently-transfected 293T cells C) Representative flow cytometry plots showing the frequency of DENV-1 RVP infected cells within the DC-SIGN, CR261 tmIgG, VDB33 tmIgA, VDB33 tmIgG, and VDB33 tmIgG positive 293T cells 24 hours after infection D) Quantification of DENV-1 RVP infected cells within the DC-SIGN, CR261 tmIgG, VDB33 tmIgA, VDB33 tmIgG, and VDB33 tmIgG positive 293T cells 24 hours after infection (PDF) [file ppat.1012683.s002.pdf]

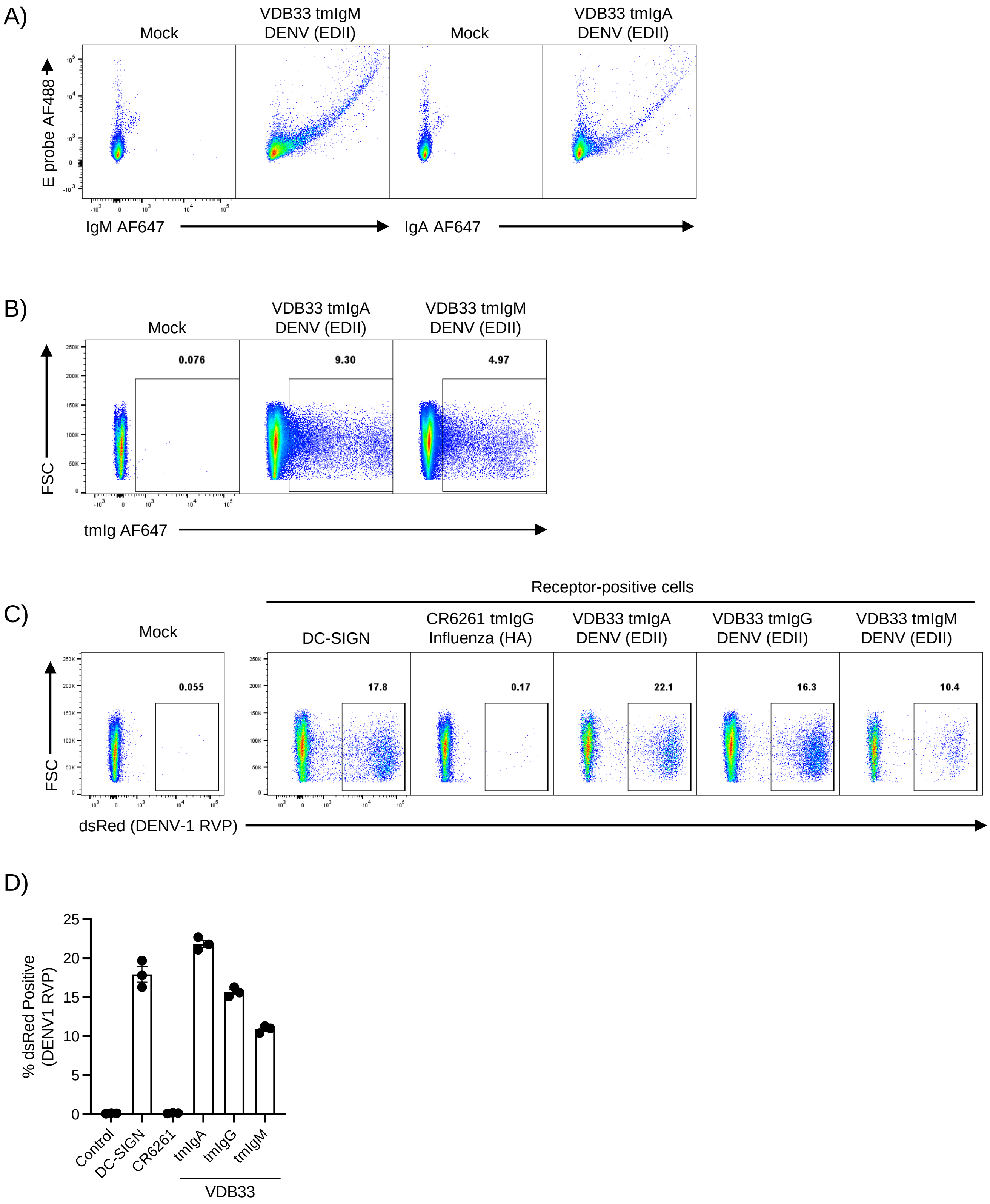

S2 Fig. Gating scheme and DENV-1 RVP infection of tmIgM and tmIgA transfected 293T cells A) Expression and DENV E protein binding activity of VDB33 tmIgM and tmIgA expression constructs. **B)** Expression and gating of tmIgM and tmIgA expression in transiently-transfected 293T cells **C)** Representative flow cytometry plots showing the frequency of DENV-1 RVP infected cells within the DC-SIGN, CR261 tmIgG, VDB33 tmIgA, VDB33 tmIgG, and VDB33 tmIgG positive 293T cells 24 hours after infection **D)** Quantification of DENV-1 RVP infected cells within the DC-SIGN, CR261 tmIgG, VDB33 tmIgA, VDB33 tmIgG, and VDB33 tmIgG positive 293T cells 24 hours after infection
