# Supplementary material for: B cell receptor dependent enhancement of dengue virus infection
Source: PLoS Pathog. 2024 Oct 31;20(10):e1012683. doi: 10.1371/journal.ppat.1012683 (PMC11556684; doi:10.1371/journal.ppat.1012683)
Supplement: S3 Fig — A) Binding of the indicated IgG isotype mAbs to recombinant/purified HCMV gH protein as quantified by ELISA. B) Expression and gating of tmIgG and PDGFRa expression in transiently-transfected 293T cells C) Representative flow cytometry plots showing the frequency of HCMV SV40-GFP infected cells within the receptor-positive gate of tmIgG and PDGFRa transfected 293T cells 24hrs after virus inoculation. Cells infected at an MOI of 1 D) Quantification of HCMV SV40-GFP infected cells within the receptor-negative gate of tmIgG and PDGFRa in transfected 293T cells 24hrs after virus inoculation (PDF) [file ppat.1012683.s003.pdf]

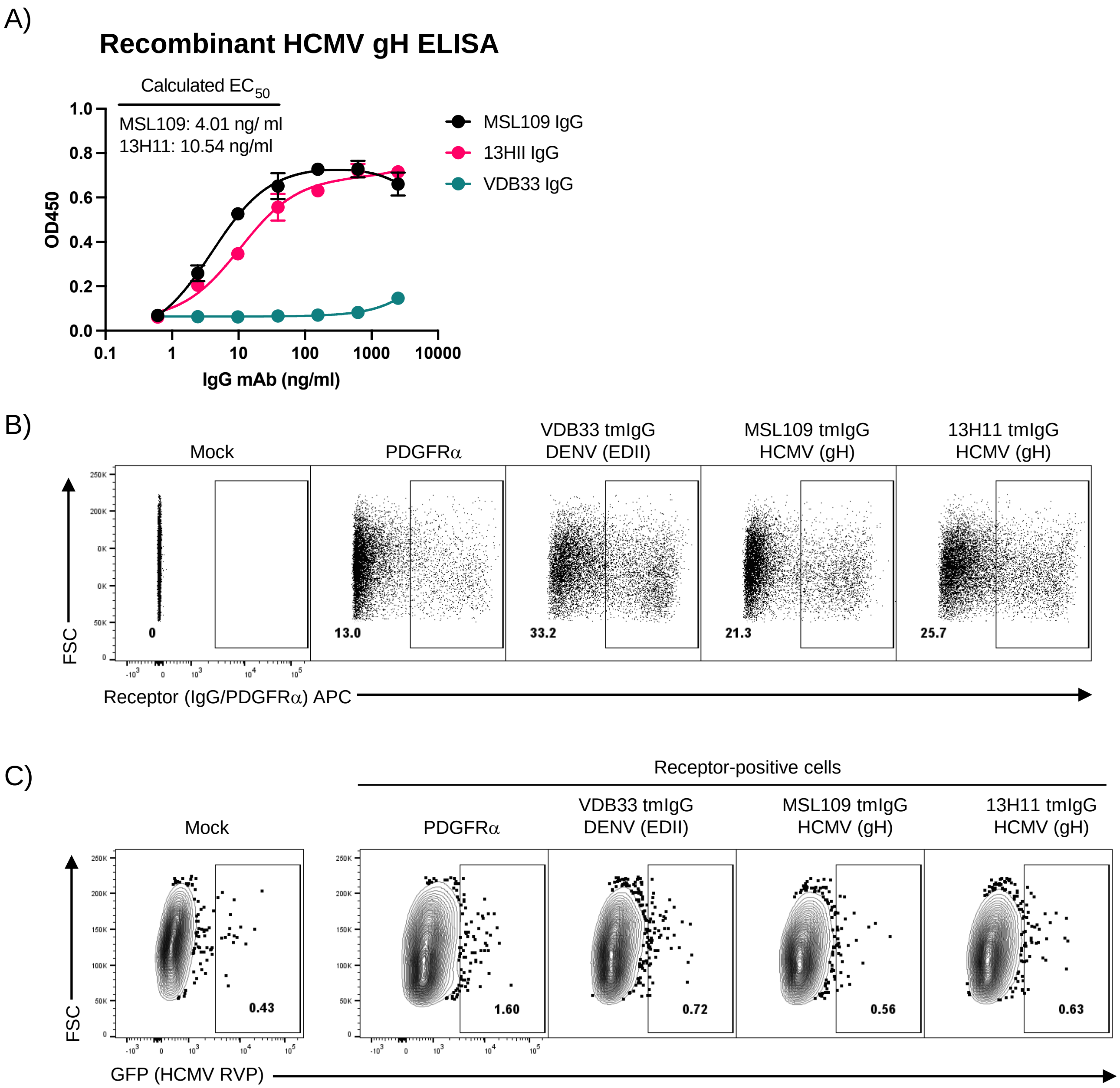

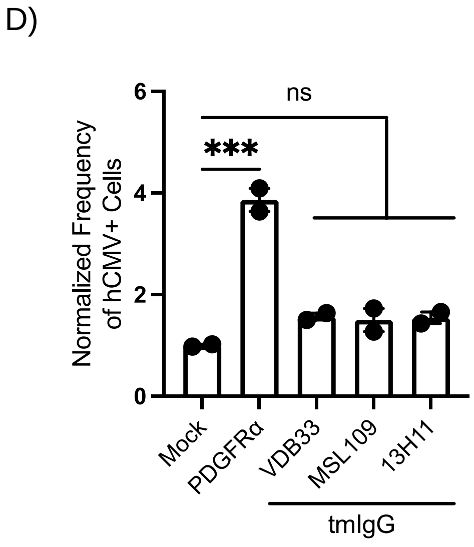

S3 Fig. Expression of HCMV specific tmIgG. A) Binding of the indicated IgG isotype mAbs to recombinant/purified HCMV gH protein as quantified by ELISA. **B)** Expression and gating of tmIgG and PDGFRα expression in transiently-transfected 293T cells **C)** Representative flow cytometry plots showing the frequency of HCMV SV40-GFP infected cells within the receptor-positive gate of tmIgG and PDGFRα transfected 293T cells 24hrs after virus inoculation. Cells infected at an MOI of 1 **D)** Quantification of HCMV SV40-GFP infected cells within the receptor-negative gate of tmIgG and PDGFRα in transfected 293T cells 24hrs after virus inoculation
