# Supplementary material for: B cell receptor dependent enhancement of dengue virus infection
Source: PLoS Pathog. 2024 Oct 31;20(10):e1012683. doi: 10.1371/journal.ppat.1012683 (PMC11556684; doi:10.1371/journal.ppat.1012683)
Supplement: S4 Fig — A) Schematic representation of 7B9 and 2F3 cell line generation and maintenance. Created in BioRender. B) Virus binding ELISA data for mAbs expressed by 7B9 and C) 2F3 cell lines. D) DENV and ZIKV neutralization profiles of mAb expressed by cell line 7B9. E) Gating scheme for flow cytometry analysis of DENV-infected 7B9 and 2F3 cell lines. F) Representative flow cytometry plots showing the frequency of DENV-infected 7B9 and 2F3 cell lines after DENV-2 exposure. (PDF) [file ppat.1012683.s004.pdf]

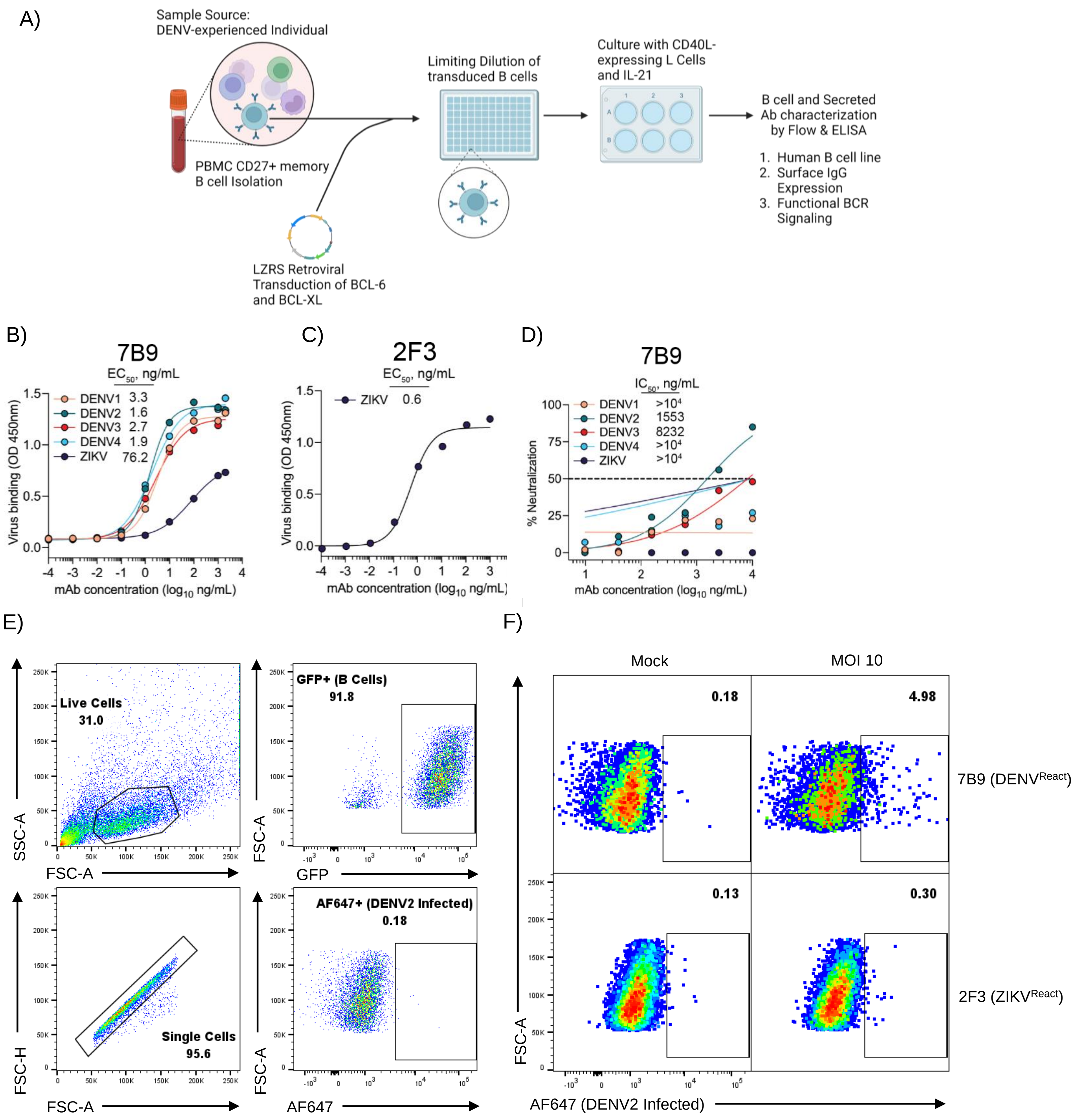

S4 Fig. Characterization and DENV-infection of 7B9 and 2F3 B cell lines. A) Schematic representation of 7B9 and 2F3 cell line generation and maintenance. Created in BioRender. **B)** Virus binding ELISA data for mAbs expressed by 7B9 and C) 2F3 cell lines. **D)** DENV and ZIKV neutralization profiles of mAb expressed by cell line 7B9. **E)** Gating scheme for flow cytometry analysis of DENV-infected 7B9 and 2F3 cell lines. **F)** Representative flow cytometry plots showing the frequency of DENV-infected 7B9 and 2F3 cell lines after DENV-2 exposure.
