# Supplementary material for: B cell receptor dependent enhancement of dengue virus infection
Source: PLoS Pathog. 2024 Oct 31;20(10):e1012683. doi: 10.1371/journal.ppat.1012683 (PMC11556684; doi:10.1371/journal.ppat.1012683)
Supplement: S5 Fig — A) Violin plots indicating number of features (nFeatures) and percentage mitochondrial content (percent mt) pre-filtering of scRNAseq data. B) Integrated UMAP projection of cells derived from all conditions prior to subsetting for removal of CD40 ligand positive feeder cell population, and C) Feature plot indicating CD40 ligand (CD40LG) expression, highlighting the feeder cell population (cluster 3) which was removed for subsequent analysis. D) UMAP projections of scRNAseq data indicating Seurat clusters, E) Sample origin, and F) Cell cycle phase. G) Feature plot indicating DENV (+) and (-) sense RNA expression. H) Imputed cell labelling of infected (positive for both DENV positive and negative sense RNA) and uninfected control/bystander cell populations. I) Dot plot highlighting selectively upregulated and downregulated DEGs in infected (expressing both DENV (+) and (-) RNA) compared to uninfected control cells (lacking both DENV (+) and (-) RNA expression). Average expression and percent of cells expressing a given transcript are indicated. J) Ingenuity Pathway Analysis (IPA) Graphical summary of predicted pathway enrichment in infected cells compared to uninfected controls based on differential gene expression between infected (expressing both DENV (+) and (-) RNA) compared to uninfected control cells (lacking both DENV (+) and (-) RNA expression). (PDF) [file ppat.1012683.s005.pdf]

Supplemental Figure 5

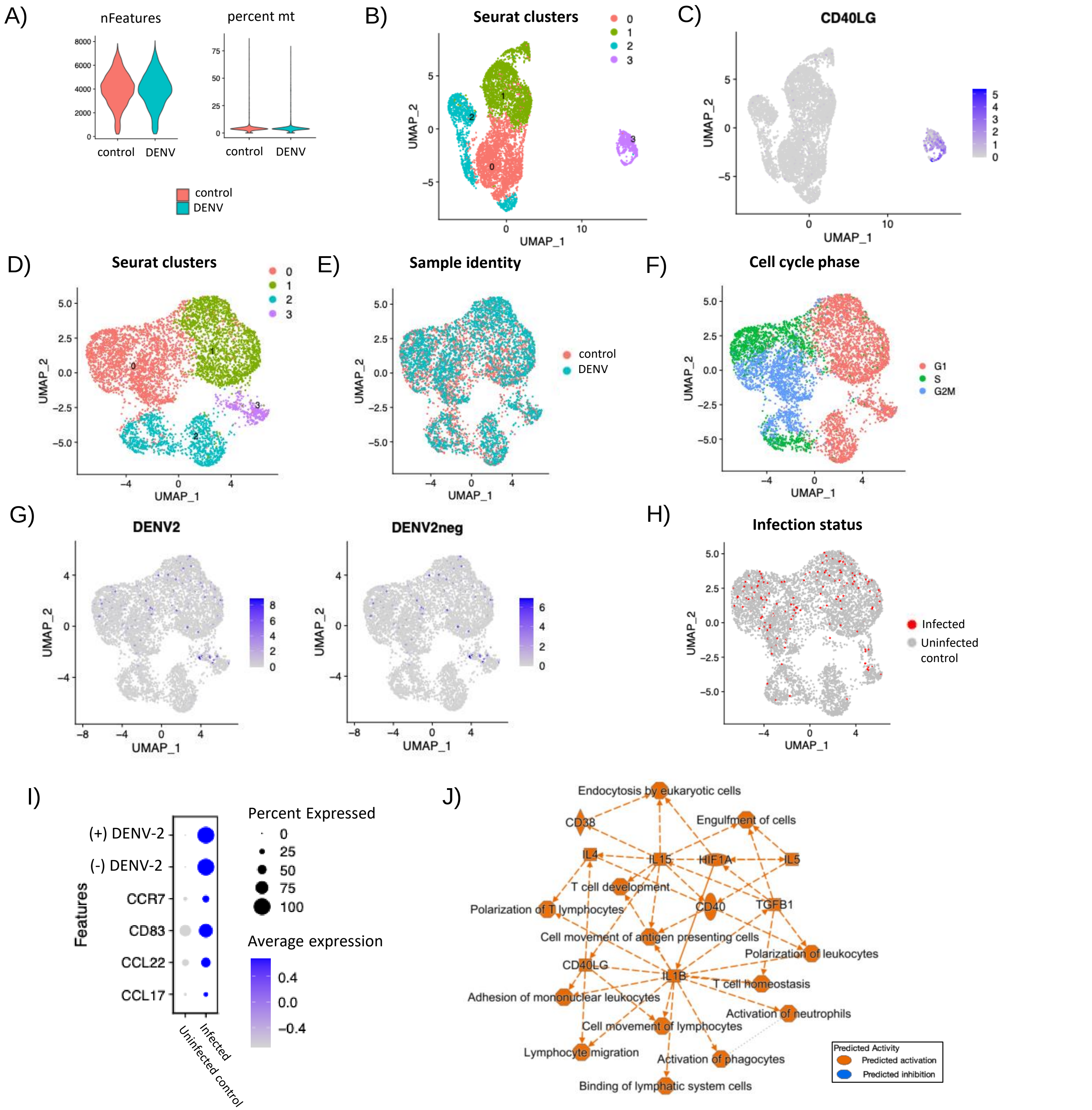

S5 Fig. Quality control metrics and integrated UMAP projections of scRNA seq data. **A)** Violin plots indicating number of features (nFeatures) and percentage mitochondrial content (percent mt) pre-filtering of scRNAseq data. **B)** Integrated UMAP projection of cells derived from all conditions prior to subsetting for removal of CD40 ligand positive feeder cell population, and **C)** Feature plot indicating CD40 ligand (CD40LG) expression, highlighting the feeder cell population (cluster 3) which was removed for subsequent analysis. **D)** UMAP projections of scRNAseq data indicating Seurat clusters, **E)** Sample origin, and **F)** Cell cycle phase. **G**) Feature plot indicating DENV (+) and (-) sense RNA expression. **H)** Imputed cell labelling of infected (positive for both DENV positive and negative sense RNA) and uninfected control/bystander cell populations. **I)** Dot plot highlighting selectively upregulated and downregulated DEGs in infected (expressing both DENV (+) and (-) RNA) compared to uninfected control cells (lacking both DENV (+) and (-) RNA expression). Average expression and percent of cells expressing a given transcript are indicated. **J**) Ingenuity Pathway Analysis (IPA) Graphical summary of predicted pathway enrichment in infected cells compared to uninfected controls based on differential gene expression between infected (expressing both DENV (+) and (-) RNA) compared to uninfected control cells (lacking both DENV (+) and (-) RNA expression).
