# Supplementary material for: B cell receptor dependent enhancement of dengue virus infection
Source: PLoS Pathog. 2024 Oct 31;20(10):e1012683. doi: 10.1371/journal.ppat.1012683 (PMC11556684; doi:10.1371/journal.ppat.1012683)
Supplement: S6 Fig — A) Schematic representation of DENV/Ig/BCR crosslinking assay. Created in BioRender. B) Representative flow cytometry plot showing the frequency of DENV-4 infected Raji cells under the indicated culture conditions. Detection of DENV-infected cells was performed by staining fixed/permeabilized cells with a FITC-conjugated 4G2 antibody. C) Quantification of DENV-4 infected Raji cells under the indicated culture conditions. D) Gating scheme for B cell infection analysis utilizing the DENV/Ig/BCR crosslinking assay. (PDF) [file ppat.1012683.s006.pdf]

Supplemental Figure 6

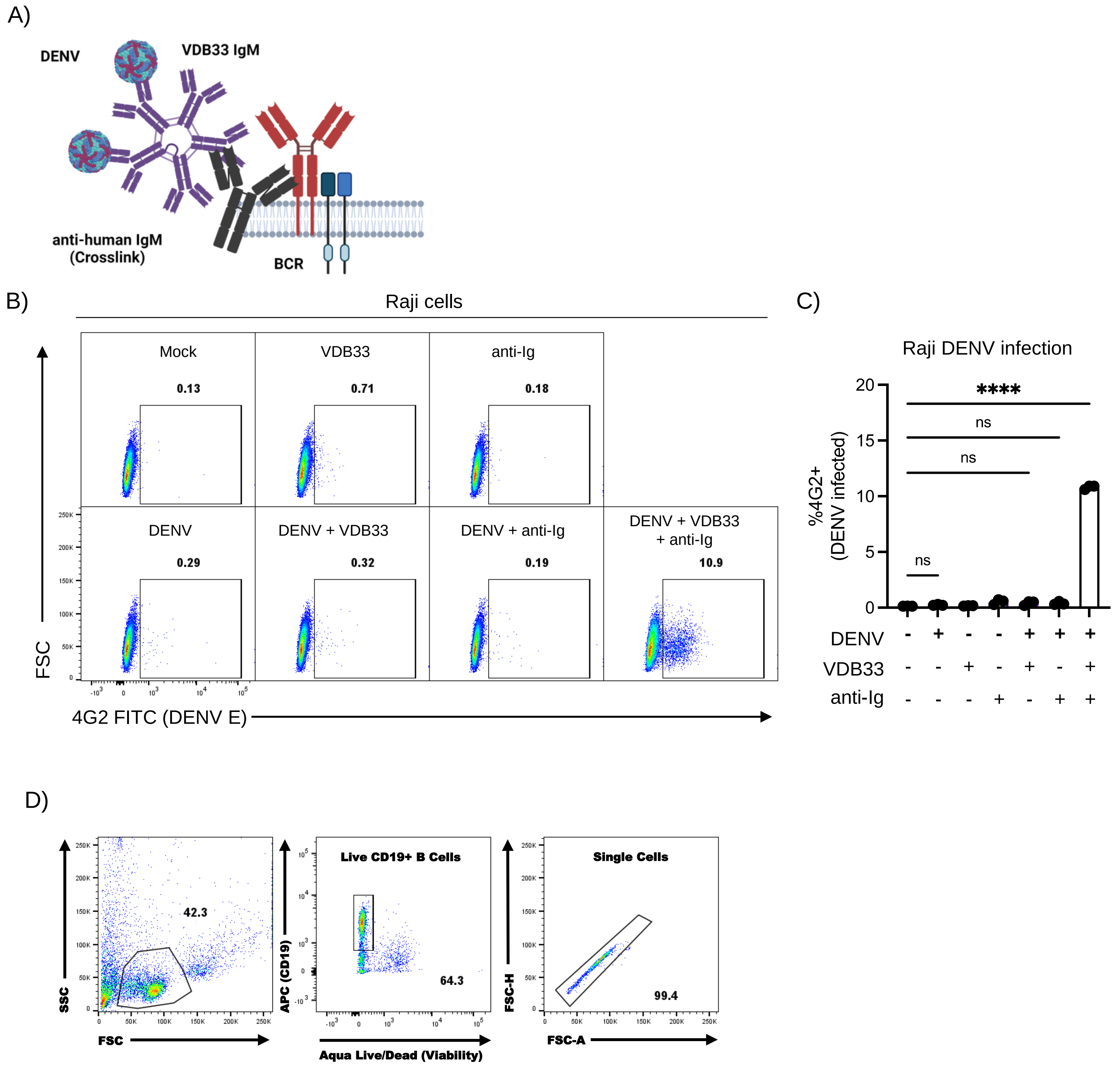

S6 Fig. DENV infection of polyclonal B cells by BCR/DENV cross-linking. A) Schematic representation of DENV/Ig/BCR crosslinking assay. Created in BioRender. **B**) Representative flow cytometry plot showing the frequency of DENV-4 infected Raji cells under the indicated culture conditions. Detection of DENV-infected cells was performed by staining fixed/permeabilized cells with a FITC-conjugated 4G2 antibody. **C)** Quantification of DENV-4 infected Raji cells under the indicated culture conditions. **D)** Gating scheme for B cell infection analysis utilizing the DENV/Ig/BCR crosslinking assay.
