# Supplementary figures and images for: B cell receptor dependent enhancement of dengue virus infection
Source: PLoS Pathog. 2024 Oct 31;20(10):e1012683. doi: 10.1371/journal.ppat.1012683 (PMC11556684; doi:10.1371/journal.ppat.1012683)

A)

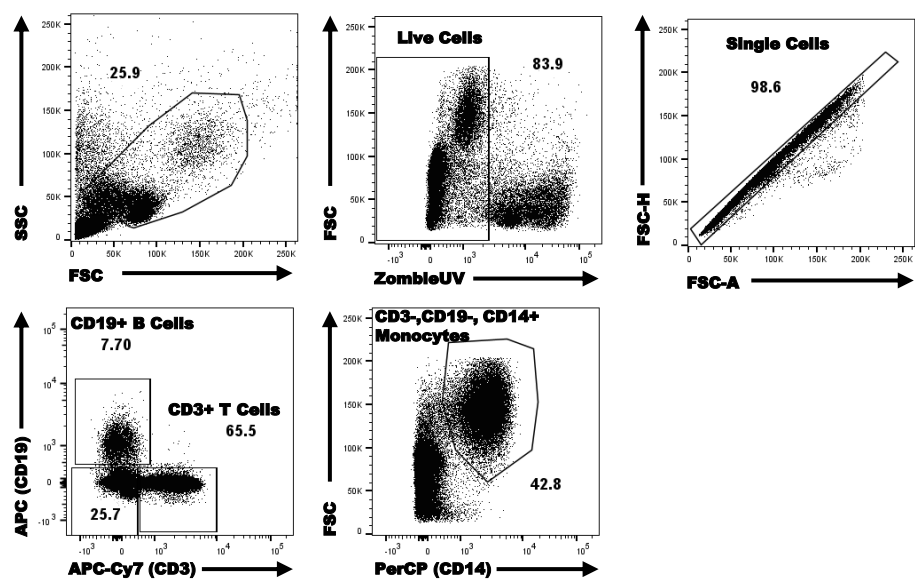

S7 Fig. Gating scheme for DHIM-3 PBMC analysis

Supplement: S7 Fig — (PDF) [file ppat.1012683.s007.pdf]
