# Supplementary material for: B cell receptor dependent enhancement of dengue virus infection
Source: PLoS Pathog. 2024 Oct 31;20(10):e1012683. doi: 10.1371/journal.ppat.1012683 (PMC11556684; doi:10.1371/journal.ppat.1012683)
Supplement: S1 Table — (DOCX) [file ppat.1012683.s008.docx]

**S1 Table.** Quality control metrics for scRNAseq data.

| **Sample** | **Number of reads** | **Estimated number of cells** | **Mean reads per cell** | **Median genes per cell** | **Median UMI counts per cell** |
| --- | --- | --- | --- | --- | --- |
| **Control** | 239 120 923 | 3 419 | 69 939 | 4 004 | 17 248 |
| **DENV-2** | 293 412 699 | 3 274 | 89 619 | 3 933 | 17 115 |
