# Supplementary material for: B cell receptor dependent enhancement of dengue virus infection
Source: PLoS Pathog. 2024 Oct 31;20(10):e1012683. doi: 10.1371/journal.ppat.1012683 (PMC11556684; doi:10.1371/journal.ppat.1012683)
Supplement: S2 Table — (DOCX) [file ppat.1012683.s009.docx]

**S2 Table.** Infected cell characteristics across culture conditions.

| **Sample** | Total cells  n | (+) RNA only  n (%) | (-) RNA only  n (%) | (+) and (-) RNA  n (%) | **Percentage productively infected cells (%)** |
| --- | --- | --- | --- | --- | --- |
| **Control** | 3089 | 4 (0) | 0 (0) | 3 (0) | **0** |
| **DENV** | 2874 | 323 (11.2) | 46 (1.6) | 158 (5.5) | **5.5** |
