# Supplementary material for: B cell receptor dependent enhancement of dengue virus infection
Source: PLoS Pathog. 2024 Oct 31;20(10):e1012683. doi: 10.1371/journal.ppat.1012683 (PMC11556684; doi:10.1371/journal.ppat.1012683)
Supplement: S3 Table — (DOCX) [file ppat.1012683.s010.docx]

**S3 Table.** Differentially expressed genes, DENV-2 infected cells and uninfected mock cells

| Gene | p_val | avg_log2FC | pct.1 | pct.2 | p_val_adj |
| --- | --- | --- | --- | --- | --- |
| DENV2 | 0 | 7.78946458 | 1 | 0 | 0 |
| DENV2neg | 0 | 5.64984623 | 1 | 0 | 0 |
| RPS29 | 7.25999011232681e-83 | 1.35628816 | 0.981 | 0.986 | 1.46564680387654e-78 |
| RPS21 | 1.4044602476356e-57 | 0.57383511 | 0.987 | 0.993 | 2.83532434792676e-53 |
| MT-ATP8 | 3.02340174175545e-54 | 1.01227813 | 0.994 | 0.97 | 6.10364343625591e-50 |
| RPL37A | 1.16825240324941e-53 | 0.53082581 | 0.981 | 0.994 | 2.35846795167992e-49 |
| ATP5ME | 1.74628912311265e-53 | 0.88281685 | 0.962 | 0.914 | 3.52540848173981e-49 |
| RPS27 | 1.52768057031352e-48 | 0.56638913 | 0.994 | 0.993 | 3.08408153534894e-44 |
| MT-ND3 | 1.39160781456075e-47 | 0.73113374 | 0.994 | 0.987 | 2.80937785603525e-43 |
| RPL38 | 1.39618780958835e-46 | 0.61665388 | 0.994 | 0.983 | 2.81862394999695e-42 |
| RPL37 | 2.10120092693786e-46 | 0.46669258 | 0.987 | 0.993 | 4.24190443130216e-42 |
| RMRP | 3.98916886228975e-46 | 0.86657363 | 0.728 | 0.251 | 8.05333409919055e-42 |
| NDUFB1 | 4.39891030636231e-38 | 0.71358662 | 0.949 | 0.863 | 8.88052012648424e-34 |
| RPL26 | 1.08898352981008e-34 | -0.5123385 | 0.968 | 0.99 | 2.19843994998059e-30 |
| UBD | 5.77634064960661e-33 | 0.34065402 | 0.487 | 0.141 | 1.16612765034258e-28 |
| ATP5F1E | 9.27021771196101e-33 | 0.45398847 | 0.968 | 0.988 | 1.87147155169069e-28 |
| RPS15A | 2.4407716955149e-28 | -0.4029856 | 0.987 | 0.994 | 4.92742989890547e-24 |
| NME2 | 1.06047926068114e-27 | 0.52578956 | 0.949 | 0.863 | 2.14089553146308e-23 |
| OAZ1 | 1.56817937998613e-24 | -0.4862034 | 0.981 | 0.985 | 3.165840532316e-20 |
| RPS28 | 1.27749003607066e-22 | 0.33160919 | 0.994 | 0.994 | 2.57899688481945e-18 |
| COX16 | 3.40763827400769e-22 | 0.46417728 | 0.867 | 0.654 | 6.87934014756672e-18 |
| ROMO1 | 9.06172504820304e-22 | 0.46921768 | 0.949 | 0.945 | 1.82938105273123e-17 |
| RPL36 | 1.61912291452079e-21 | 0.29645018 | 0.994 | 0.992 | 3.26868533983457e-17 |
| MIF | 2.06282658931158e-21 | 0.42464314 | 0.975 | 0.984 | 4.16443431850221e-17 |
| ALDOA | 2.80330944601422e-21 | 0.48325969 | 0.975 | 0.937 | 5.6593211096135e-17 |
| EIF5B | 5.69182820695662e-21 | 0.51527792 | 0.956 | 0.89 | 1.1490662784204e-16 |
| RPL39 | 1.11630036014419e-20 | 0.36510245 | 0.981 | 0.992 | 2.25358716705909e-16 |
| NDUFA3 | 3.76229184241984e-20 | 0.45464489 | 0.924 | 0.81 | 7.59531477147716e-16 |
| PTPRCAP | 4.82252370572321e-20 | 0.26526699 | 0.62 | 0.285 | 9.73571085711401e-16 |
| MTRNR2L12 | 1.60868694252421e-19 | -0.6386891 | 0.994 | 0.992 | 3.24761719956788e-15 |
| YWHAE | 1.29109630290322e-18 | 0.38938592 | 0.975 | 0.97 | 2.60646521630102e-14 |
| RPSA | 2.78908460063567e-18 | -0.2891266 | 0.987 | 0.989 | 5.63060399176328e-14 |
| DDT | 1.49190884199643e-17 | -0.4452174 | 0.873 | 0.929 | 3.01186557022239e-13 |
| ARPC1B | 2.75616116535174e-17 | 0.36627286 | 0.873 | 0.658 | 5.56413816061209e-13 |
| NEDD8 | 1.15235661387404e-16 | -0.4267738 | 0.88 | 0.928 | 2.32637753208891e-12 |
| RPS3A | 2.22088635006074e-16 | -0.2973643 | 0.987 | 0.996 | 4.48352536350263e-12 |
| TMA7 | 1.0108239307923e-15 | 0.33225826 | 0.994 | 0.985 | 2.0406513514835e-11 |
| EIF3J | 9.70854194640473e-15 | 0.38694512 | 0.861 | 0.659 | 1.95996044814019e-10 |
| RPL34 | 2.71187397446657e-14 | 0.28326956 | 0.987 | 0.994 | 5.47473117965312e-10 |
| TOMM5 | 3.61786651933212e-14 | 0.43725135 | 0.943 | 0.874 | 7.30374892922769e-10 |
| PSMA6 | 6.41696007303664e-14 | 0.37102156 | 0.911 | 0.757 | 1.29545589954464e-09 |
| ATP5MD | 1.00706280856607e-13 | 0.33729591 | 0.968 | 0.957 | 2.03305839793318e-09 |
| SLIRP | 1.2755346583616e-13 | 0.42725472 | 0.956 | 0.93 | 2.5750493683004e-09 |
| CMSS1 | 2.74354398831438e-13 | 0.25976057 | 0.677 | 0.429 | 5.53866660360908e-09 |
| PET100 | 7.84949360704804e-13 | 0.3116727 | 0.88 | 0.712 | 1.58465576939086e-08 |
| RUNX3 | 7.85331975575218e-13 | 0.37087152 | 0.886 | 0.721 | 1.58542819229125e-08 |
| DDX1 | 8.27141834192928e-13 | 0.34850161 | 0.823 | 0.646 | 1.66983393486868e-08 |
| MIR155HG | 1.47645514321639e-12 | 0.53260008 | 0.797 | 0.545 | 2.98066764312524e-08 |
| TNFAIP2 | 1.7574352966517e-12 | 0.60251258 | 0.937 | 0.82 | 3.54791037688044e-08 |
| ABCF1 | 3.57749450564282e-12 | 0.37302376 | 0.899 | 0.75 | 7.22224590799173e-08 |
| SAMSN1 | 5.97179491289947e-12 | 0.43817783 | 0.804 | 0.575 | 1.20558595701614e-07 |
| NDUFA11 | 7.30134428300667e-12 | -0.3284874 | 0.785 | 0.842 | 1.47399538385339e-07 |
| PA2G4 | 8.73064851011436e-12 | 0.44609962 | 0.949 | 0.928 | 1.76254332122189e-07 |
| METAP2 | 1.25492811923672e-11 | 0.3386349 | 0.911 | 0.811 | 2.53344888711509e-07 |
| EIF2S2 | 2.25054359597641e-11 | 0.33105807 | 0.943 | 0.91 | 4.54339741155719e-07 |
| COX6A1 | 2.27161015403552e-11 | -0.2942879 | 0.981 | 0.982 | 4.58592657896692e-07 |
| NARS | 2.62334462180443e-11 | 0.26499175 | 0.829 | 0.606 | 5.29600812249878e-07 |
| PPA1 | 2.71720292700571e-11 | 0.30753662 | 0.968 | 0.959 | 5.48548926903913e-07 |
| PPDPF | 4.02509472606887e-11 | -0.3969425 | 0.924 | 0.945 | 8.12586123298784e-07 |
| RPS4X | 5.87743488249973e-11 | -0.2558316 | 0.994 | 0.995 | 1.18653655407905e-06 |
| S100A4 | 7.41867516922651e-11 | 0.51334713 | 0.772 | 0.577 | 1.49768214316345e-06 |
| H3F3A | 8.32096806568795e-11 | -0.2919772 | 0.975 | 0.991 | 1.67983703310108e-06 |
| MRPL23 | 1.16849639390254e-10 | -0.2976701 | 0.405 | 0.586 | 2.35896052001044e-06 |
| TAF11 | 1.32539947611917e-10 | 0.25587334 | 0.759 | 0.526 | 2.67571646238939e-06 |
| PPP2CA | 1.3578764262815e-10 | -0.3642489 | 0.658 | 0.768 | 2.7412809293771e-06 |
| H3F3B | 1.84321826352206e-10 | -0.3096507 | 0.987 | 0.993 | 3.72108903039833e-06 |
| MT-ATP6 | 2.7545535464703e-10 | -0.32063 | 1 | 0.994 | 5.56089269961423e-06 |
| DENR | 6.15790294291304e-10 | 0.27764285 | 0.886 | 0.756 | 1.24315744611528e-05 |
| SLC25A5 | 9.78785086703137e-10 | -0.3025437 | 0.943 | 0.979 | 1.97597133303629e-05 |
| NDUFA1 | 1.21867464662305e-09 | 0.27854151 | 0.968 | 0.941 | 2.46026037660262e-05 |
| MT-ND2 | 1.36006841469263e-09 | 0.30662285 | 1 | 0.985 | 2.74570611558149e-05 |
| AL138963.3 | 2.63463105579513e-09 | -0.5445581 | 0.741 | 0.804 | 5.31879317543921e-05 |
| CCR7 | 3.42496449185524e-09 | 0.70978491 | 0.354 | 0.172 | 6.91431831615736e-05 |
| SRSF9 | 3.52210730495601e-09 | -0.2780663 | 0.968 | 0.966 | 7.11043022724518e-05 |
| CORO1A | 6.55693077567514e-09 | -0.3485382 | 0.949 | 0.958 | 0.00013237 |
| DPYSL2 | 8.88444384567165e-09 | 0.28215948 | 0.772 | 0.568 | 0.00017936 |
| SNRPB2 | 1.05693364179265e-08 | 0.30624269 | 0.892 | 0.797 | 0.00021337 |
| PRDX2 | 1.21359193980804e-08 | -0.3082327 | 0.949 | 0.966 | 0.000245 |
| ERH | 2.13954803762635e-08 | -0.2748098 | 0.962 | 0.978 | 0.00043193 |
| IGLC3 | 3.01192707291274e-08 | 0.40509046 | 0.93 | 0.752 | 0.00060805 |
| FNBP1 | 3.04446929763137e-08 | 0.29256032 | 0.905 | 0.813 | 0.00061462 |
| NAPSA | 4.76436719908735e-08 | -0.3482562 | 0.538 | 0.639 | 0.00096183 |
| ARPC5 | 7.09231545556128e-08 | -0.2946798 | 0.968 | 0.979 | 0.0014318 |
| MKNK2 | 1.2468912670071e-07 | 0.3506819 | 0.861 | 0.722 | 0.00251722 |
| CD83 | 1.31044635033675e-07 | 0.41216608 | 0.804 | 0.647 | 0.00264553 |
| HLA-C | 1.34908219653009e-07 | -0.3426838 | 0.956 | 0.966 | 0.00272353 |
| DDX21 | 1.74955084302371e-07 | 0.33254476 | 0.918 | 0.9 | 0.00353199 |
| PPP1CA | 1.85579640066829e-07 | -0.2562549 | 0.937 | 0.949 | 0.00374648 |
| ATP6V1G1 | 1.88510744852717e-07 | -0.3312708 | 0.943 | 0.952 | 0.00380565 |
| GPR157 | 1.9375995077652e-07 | 0.27679599 | 0.437 | 0.261 | 0.00391163 |
| LCP1 | 2.25436168594684e-07 | 0.2871279 | 0.987 | 0.985 | 0.00455111 |
| MALAT1 | 2.26555259303704e-07 | 0.33674625 | 1 | 0.995 | 0.0045737 |
| SKP1 | 2.3382582128986e-07 | -0.2617133 | 0.943 | 0.95 | 0.00472048 |
| CCNI | 2.5862007400521e-07 | -0.274419 | 0.937 | 0.954 | 0.00522102 |
| IGKV3-15 | 2.64167659909777e-07 | -1.3226319 | 0.082 | 0.252 | 0.00533302 |
| PDCD11 | 2.80322090233665e-07 | 0.2694218 | 0.728 | 0.547 | 0.00565914 |
| CCNG1 | 3.13780313320918e-07 | -0.3096497 | 0.918 | 0.908 | 0.0063346 |
| WDR83OS | 3.33462018465226e-07 | -0.2857171 | 0.924 | 0.933 | 0.00673193 |
| TMEM14B | 4.34447239092933e-07 | -0.2755085 | 0.842 | 0.871 | 0.00877062 |
| IGKV3D-11 | 5.36806135548254e-07 | -1.1740944 | 0.082 | 0.25 | 0.01083704 |
| DYNLL1 | 5.37537015403036e-07 | -0.3371743 | 0.93 | 0.964 | 0.0108518 |
| LIMD2 | 5.69800488403979e-07 | -0.2836384 | 0.975 | 0.963 | 0.01150313 |
| ANP32B | 6.29284332714697e-07 | -0.2804014 | 0.968 | 0.968 | 0.01270399 |
| IGKV3-11 | 7.62445584680517e-07 | -1.5906087 | 0.095 | 0.256 | 0.01539225 |
| TNNI1 | 8.02541581968897e-07 | 0.3400626 | 0.519 | 0.317 | 0.01620171 |
| PLEC | 8.52500839120461e-07 | 0.35477235 | 0.753 | 0.591 | 0.01721029 |
| PSMD9 | 9.01146687812747e-07 | -0.2714013 | 0.582 | 0.69 | 0.01819235 |
| EIF3H | 9.90101493396531e-07 | -0.2639272 | 0.956 | 0.967 | 0.01998817 |
| CD44 | 9.91814559212962e-07 | 0.38893039 | 0.241 | 0.114 | 0.02002275 |
| IGKC | 1.15333776199585e-06 | -1.2112693 | 0.114 | 0.276 | 0.02328358 |
| SCD | 1.87330420715242e-06 | 0.26534152 | 0.854 | 0.761 | 0.03781827 |
| CCL17 | 1.88548054439542e-06 | 0.59604529 | 0.19 | 0.08 | 0.03806408 |
| FTL | 1.90599002852873e-06 | -0.3101528 | 0.981 | 0.994 | 0.03847813 |
| IGHV5-51 | 1.99311771255404e-06 | -1.49403 | 0.095 | 0.254 | 0.04023706 |
| FLNA | 2.02226636035301e-06 | 0.31834453 | 0.911 | 0.87 | 0.04082551 |
| TIMP1 | 2.02410921494935e-06 | 0.30435698 | 0.924 | 0.851 | 0.04086272 |
| CCL22 | 2.14564901093193e-06 | 0.60350096 | 0.532 | 0.345 | 0.04331636 |
| HYOU1 | 2.15838041353401e-06 | 0.25599756 | 0.829 | 0.695 | 0.04357338 |
