# Supplementary material for: B cell receptor dependent enhancement of dengue virus infection
Source: PLoS Pathog. 2024 Oct 31;20(10):e1012683. doi: 10.1371/journal.ppat.1012683 (PMC11556684; doi:10.1371/journal.ppat.1012683)
Supplement: S4 Table — (DOCX) [file ppat.1012683.s011.docx]

**S4 Table.** Differentially expressed pathways between DENV-2 infected 7B9 cells and uninfected mock cells

| **Ingenuity Canonical Pathways** | **-log(p-value)** | **Ratio** | **z-score** |
| --- | --- | --- | --- |
| Eukaryotic Translation Initiation | 23.3 | 0.156 | 1.606 |
| Response of EIF2AK4 (GCN2) to amino acid deficiency | 19.6 | 0.155 | 1.5 |
| EIF2 Signaling | 19.3 | 0.087 | 1.667 |
| Nonsense-Mediated Decay (NMD) | 18.7 | 0.137 | 1 |
| Eukaryotic Translation Termination | 18.6 | 0.16 | 1.291 |
| Eukaryotic Translation Elongation | 18.5 | 0.158 | 1.291 |
| Selenoamino acid metabolism | 17.7 | 0.14 | 1.291 |
| SRP-dependent cotranslational protein targeting to membrane | 17.2 | 0.13 | 1.291 |
| Major pathway of rRNA processing in the nucleolus and cytosol | 16.8 | 0.0914 | 1.698 |
| Electron transport, ATP synthesis, and heat production by uncoupling proteins | 12.1 | 0.0938 | 1.732 |
| Regulation of eIF4 and p70S6K Signaling | 10.2 | 0.0652 | #NUM! |
| Oxidative Phosphorylation | 9.92 | 0.0893 | 1.265 |
| mTOR Signaling | 9.44 | 0.0561 | #NUM! |
| Sirtuin Signaling Pathway | 6.92 | 0.0378 | -1.89 |
| Coronavirus Pathogenesis Pathway | 6.31 | 0.0441 | -0.333 |
| Granzyme A Signaling | 5.89 | 0.0811 | -1.633 |
| Neutrophil Extracellular Trap Signaling Pathway | 5.56 | 0.0275 | 0.905 |
| Mitochondrial Dysfunction | 5.31 | 0.0291 | -1.265 |
| Cristae formation | 4.9 | 0.129 | 1 |
| Binding and Uptake of Ligands by Scavenger Receptors | 4.85 | 0.0536 | -0.816 |
| rRNA processing | 4.84 | 0.125 | #NUM! |
| Signaling by the B Cell Receptor (BCR) | 4.82 | 0.0412 | -1.134 |
| Hematoma Resolution Signaling Pathway | 4.54 | 0.031 | 1.414 |
| Fc epsilon receptor (FCERI) signaling | 4.29 | 0.034 | -1.134 |
| HIPPO signaling | 4.26 | 0.0575 | #NUM! |
| C-type lectin receptors (CLRs) | 4.21 | 0.0414 | 0 |
| Fcgamma receptor (FCGR) dependent phagocytosis | 4.02 | 0.0382 | -0.816 |
| Parkinson's Signaling Pathway | 4.01 | 0.0261 | -1.414 |
| Eumelanin Biosynthesis | 3.89 | 0.5 | #NUM! |
| Iron uptake and transport | 3.81 | 0.069 | -2 |
| NIK-->noncanonical NF-kB signaling | 3.75 | 0.0667 | -1 |
| Mitotic G2-G2/M phases | 3.46 | 0.0302 | -0.816 |
| Neutrophil degranulation | 3.41 | 0.0189 | 0.333 |
| Glucocorticoid Receptor Signaling | 3.4 | 0.0172 | #NUM! |
| Cell surface interactions at the vascular wall | 3.33 | 0.0284 | 0 |
| TP53 Regulates Metabolic Genes | 3.11 | 0.0455 | 0 |
| Degradation of beta-catenin by the destruction complex | 3.04 | 0.0435 | -1 |
| ABC-family proteins mediated transport | 2.89 | 0.0396 | 1 |
| Cell Cycle Checkpoints | 2.76 | 0.0221 | 0 |
| Macrophage Alternative Activation Signaling Pathway | 2.7 | 0.0263 | 1.342 |
| FAT10 Signaling Pathway | 2.62 | 0.0526 | #NUM! |
| Immunoregulatory interactions between a Lymphoid and a non-Lymphoid cell | 2.59 | 0.0248 | -1.342 |
| Metabolism of polyamines | 2.58 | 0.0508 | #NUM! |
| Estrogen Receptor Signaling | 2.51 | 0.0171 | #NUM! |
| Mitotic G1 phase and G1/S transition | 2.48 | 0.0305 | #NUM! |
| Complement cascade | 2.43 | 0.0296 | #NUM! |
| Regulation of RUNX2 expression and activity | 2.32 | 0.0411 | #NUM! |
| Glioma Invasiveness Signaling | 2.32 | 0.0411 | #NUM! |
| Mitotic Metaphase and Anaphase | 2.31 | 0.0213 | -0.447 |
| p70S6K Signaling | 2.24 | 0.0138 | #NUM! |
| Neddylation | 2.23 | 0.0203 | -0.447 |
| Epithelial Adherens Junction Signaling | 2.19 | 0.0253 | 1 |
| Signaling by NOTCH4 | 2.16 | 0.0361 | #NUM! |
| Inhibition of ARE-Mediated mRNA Degradation Pathway | 2.15 | 0.0245 | #NUM! |
| Phagosome Maturation | 2.14 | 0.0244 | #NUM! |
| Cyclins and Cell Cycle Regulation | 2.12 | 0.0349 | #NUM! |
| Regulation of mitotic cell cycle | 2.09 | 0.0341 | #NUM! |
| KEAP1-NFE2L2 pathway | 2.05 | 0.033 | #NUM! |
| Crosstalk between Dendritic Cells and Natural Killer Cells | 2.05 | 0.033 | #NUM! |
| Protein Ubiquitination Pathway | 2.04 | 0.0183 | -0.447 |
| Actin Nucleation by ARP-WASP Complex | 2.03 | 0.0323 | #NUM! |
| Transcriptional regulation by RUNX3 | 1.99 | 0.0312 | #NUM! |
| S Phase | 1.94 | 0.03 | #NUM! |
| RHO GTPases Activate WASPs and WAVEs | 1.91 | 0.0556 | #NUM! |
| Protein Kinase A Signaling | 1.9 | 0.0146 | #NUM! |
| Gene and protein expression by JAK-STAT signaling after IL-12 stimulation | 1.89 | 0.0541 | #NUM! |
| Pyrophosphate hydrolysis | 1.86 | 0.333 | #NUM! |
| TCF dependent signaling in response to WNT | 1.85 | 0.0201 | 0 |
| Regulation of TP53 Expression and Degradation | 1.85 | 0.0513 | #NUM! |
| Mitotic Prometaphase | 1.82 | 0.0197 | 0 |
| Hedgehog 'off' state | 1.8 | 0.0265 | #NUM! |
| Regulation of Actin-based Motility by Rho | 1.78 | 0.0261 | #NUM! |
| Synthesis of DNA | 1.74 | 0.0252 | #NUM! |
| ERK/MAPK Signaling | 1.74 | 0.0186 | 1 |
| Interleukin-10 signaling | 1.73 | 0.0444 | #NUM! |
| RHOGDI Signaling | 1.71 | 0.0182 | -1 |
| TCR signaling | 1.67 | 0.0238 | #NUM! |
| Interleukin-1 family signaling | 1.65 | 0.0233 | #NUM! |
| Cell Cycle: G2/M DNA Damage Checkpoint Regulation | 1.63 | 0.0392 | #NUM! |
| Response to elevated platelet cytosolic Ca2+ | 1.62 | 0.0227 | #NUM! |
| Regulation of Apoptosis | 1.6 | 0.0377 | #NUM! |
| Signaling by TGF-beta Receptor Complex | 1.6 | 0.0377 | #NUM! |
| RAC Signaling | 1.58 | 0.0219 | #NUM! |
| B Cell Development | 1.57 | 0.0123 | #NUM! |
| Iron homeostasis signaling pathway | 1.57 | 0.0217 | #NUM! |
| Intrinsic Pathway for Apoptosis | 1.57 | 0.0364 | #NUM! |
| RHO GTPases Activate Formins | 1.56 | 0.0216 | #NUM! |
| Communication between Innate and Adaptive Immune Cells | 1.52 | 0.00966 | -1 |
| Circadian Clock | 1.51 | 0.0339 | #NUM! |
| Semaphorin Signaling in Neurons | 1.48 | 0.0328 | #NUM! |
| Primary Immunodeficiency Signaling | 1.48 | 0.0328 | #NUM! |
| Cytoprotection by HMOX1 | 1.47 | 0.0323 | #NUM! |
| UFMylation Signaling Pathway | 1.47 | 0.0323 | #NUM! |
| IL-15 Signaling | 1.44 | 0.0114 | #NUM! |
| Hedgehog ligand biogenesis | 1.43 | 0.0308 | #NUM! |
| FcγRIIB Signaling in B Lymphocytes | 1.42 | 0.0113 | #NUM! |
| TNFR2 non-canonical NF-kB pathway | 1.4 | 0.0294 | #NUM! |
| Remodeling of Epithelial Adherens Junctions | 1.4 | 0.0294 | #NUM! |
| OAS antiviral response | 1.39 | 0.111 | #NUM! |
| Sucrose Degradation V (Mammalian) | 1.39 | 0.111 | #NUM! |
| Cell Cycle: G1/S Checkpoint Regulation | 1.39 | 0.029 | #NUM! |
| Huntington's Disease Signaling | 1.36 | 0.0141 | #NUM! |
| Netrin Signaling | 1.33 | 0.0173 | #NUM! |
| Cellular response to hypoxia | 1.32 | 0.0267 | #NUM! |
| Caveolar-mediated Endocytosis Signaling | 1.32 | 0.0267 | #NUM! |
| WNT/β-catenin Signaling | 1.32 | 0.0172 | #NUM! |
