# Supplementary material for: B cell receptor dependent enhancement of dengue virus infection
Source: PLoS Pathog. 2024 Oct 31;20(10):e1012683. doi: 10.1371/journal.ppat.1012683 (PMC11556684; doi:10.1371/journal.ppat.1012683)
Supplement: S5 Table — (DOCX) [file ppat.1012683.s012.docx]

**S5 Table.** Reagents for flow cytometry analysis

| **Antibody** | **Clone** | **Dilution** | **Catalog #** | **Lot #** |
| --- | --- | --- | --- | --- |
| Anti-human IgA AF647 | N/A | 1:200 | Southern Biotech, 2050-31 | G0919-V490B |
| Anti-human IgG AF647 | N/A | 1:200 | Southern Biotech, 2040-31 | B3919-M950B |
| Anti-human IgM AF647 | N/A | 1:200 | Southern Biotech, 2020-31 | D2219-R121 |
| Anti-human CD19 APC | HIB19 | 1:200 | Biolegend, 302212 | B386154 |
| Anti-human CD14 PerCP | M5E2 | 1:50 | Biolegend, 301848 | B371416 |
| Anti-human CD3 APC-Cy7 | OKT3 | 1:200 | Biolegend, 317342 | B367892 |
| Anti-human DC-SIGN | 9E9A8 | 1:200 | Biolegend, 330112 | B386835 |
| Anti-human PDGFRα | 16A1 | 1:200 | Biolegend, 323512 | B388290 |
| ZombieUV | N/A | 1:200 | Biolegend, 77474 | B385840 |
| Aqua Live/Dead | N/A | 1:500 | Invitrogen, L34957 | 2204201 |
| DENV Envelop (4G2) FITC | N/A | 4ug/mL | Envigo Bioproducts, Inc, CON004 | N/A |
| Anti-mouse IgG Fab2 AF647 | N/A | 0.4ug/mL | Cell Signaling Technology, Inc, 4410S | 17 |
| Near-IR fluorescent reactive dye | N/A | 1:1000 | Invitrogen | L34975A |
| Anti-human CD19-PerCP-Cy5.5 | HIB19 | 1:50 | Biolegend, 302230 | B402462 |
| Anti-human IgG Fc APC | M1310G05 | 1:50 | Biolegend, 410712 | B405400 |
