# Supplementary material for: B cell receptor dependent enhancement of dengue virus infection
Source: PLoS Pathog. 2024 Oct 31;20(10):e1012683. doi: 10.1371/journal.ppat.1012683 (PMC11556684; doi:10.1371/journal.ppat.1012683)
Supplement: S2 Data — (DOCX) [file ppat.1012683.s014.docx]

4E11_tmIgG1

MGWSCIILFLVATATGVHSVKLLEQSGAELVKPGASVRLSCTASGFNIKDTYMSWVKQRPEQGLEWIGRIDPANGDTKYDPKFQGKATITADTSSNTAYLHLSSLTSGDTAVYYCSRGWEGFAYWGQGTLVTVSAASTKGPSVFPLAPSSKSTSGGTAALGCLVKDYFPEPVTVSWNSGALTSGVHTFPAVLQSSGLYSLSSVVTVPSSSLGTQTYICNVNHKPSNTKVDKKVEPKSCDKTHTCPPCPAPELLGGPSVFLFPPKPKDTLMISRTPEVTCVVVDVSHEDPEVKFNWYVDGVEVHNAKTKPREEQYNSTYRVVSVLTVLHQDWLNGKEYKCKVSNKALPAPIEKTISKAKGQPREPQVYTLPPSRDELTKNQVSLTCLVKGFYPSDIAVEWESNGQPENNYKTTPPVLDSDGSFFLYSKLTVDKSRWQQGNVFSCSVMHEALHNHYTQKSLSLSPELQLEESCAEAQDGELDGLWTTITIFITLFLLSVCYSATVTFFKVKWIFSSVVDLKQTIIPDYRNMIGQGA**

4E11 Light Chain

MGWSCIILFLVATATGVHSELVMTQTPASLAVSLGQRATISCRASENVDRYGNSFMHWYQQKAGQPPKLLIYRASNLESGIPARFSGSGSRTDFTLTINPVEADDVATYFCQRSNEVPWTFGGGTKLEIKRTVAAPSVFIFPPSDEQLKSGTASVVCLLNNFYPREAKVQWKVDNALQSGNSQESVTEQDSKDSTYSLSSTLTLSKADYEKHKVYACEVTHQGLSSPVTKSFNRGEC**

VDB33_tmIgM

MGWSCIILFLVATATGVHSQLQLQASGPGLVKPSETLSLTCTVSGGSIISSSYFWGWIRQPPEKELQWLGSIFSRGNAYYNPSLKSRVTVSVDTSKNQFSLKLTSVTATDTAVYYCARLLQYKWNWLFDPWGQGTLVTVSSGSASAPTLFPLVSCENSPSDTSSVAVGCLAQDFLPDSITFSWKYKNNSDISSTRGFPSVLRGGKYAATSQVLLPSKDVMQGTDEHVVCKVQHPNGNKEKNVPLPVIAELPPKVSVFVPPRDGFFGNPRKSKLICQATGFSPRQIQVSWLREGKQVGSGVTTDQVQAEAKESGPTTYKVTSTLTIKESDWLGQSMFTCRVDHRGLTFQQNASSMCVPDQDTAIRVFAIPPSFASIFLTKSTKLTCLVTDLTTYDSVTISWTRQNGEAVKTHTNISESHPNATFSAVGEASICEDDWNSGERFTCTVTHTDLPSPLKQTISRPKGVALHRPDVYLLPPAREQLNLRESATITCLVTGFSPADVFVQWMQRGQPLSPEKYVTSAPMPEPQAPGRYFAHSILTVSEEEWNTGETYTCVVAHEALPNRVTERTVDKSTEGEVSADEEGFENLWATASTFIVLFLLSLFYSTTVTLFKVK**

VDB33_tmIgG1

MGWSCIILFLVATATGVHSQLQLQASGPGLVKPSETLSLTCTVSGGSIISSSYFWGWIRQPPEKELQWLGSIFSRGNAYYNPSLKSRVTVSVDTSKNQFSLKLTSVTATDTAVYYCARLLQYKWNWLFDPWGQGTLVTVSSASTKGPSVFPLAPSSKSTSGGTAALGCLVKDYFPEPVTVSWNSGALTSGVHTFPAVLQSSGLYSLSSVVTVPSSSLGTQTYICNVNHKPSNTKVDKKVEPKSCDKTHTCPPCPAPELLGGPSVFLFPPKPKDTLMISRTPEVTCVVVDVSHEDPEVKFNWYVDGVEVHNAKTKPREEQYNSTYRVVSVLTVLHQDWLNGKEYKCKVSNKALPAPIEKTISKAKGQPREPQVYTLPPSRDELTKNQVSLTCLVKGFYPSDIAVEWESNGQPENNYKTTPPVLDSDGSFFLYSKLTVDKSRWQQGNVFSCSVMHEALHNHYTQKSLSLSPELQLEESCAEAQDGELDGLWTTITIFITLFLLSVCYSATVTFFKVKWIFSSVVDLKQTIIPDYRNMIGQGA**

VDB33_tmIgA1

MGWSCIILFLVATATGVHSQLQLQASGPGLVKPSETLSLTCTVSGGSIISSSYFWGWIRQPPEKELQWLGSIFSRGNAYYNPSLKSRVTVSVDTSKNQFSLKLTSVTATDTAVYYCARLLQYKWNWLFDPWGQGTLVTVSSASPTSPKVFPLSLCSTQPDGNVVIACLVQGFFPQEPLSVTWSESGQGVTARNFPPSQDASGDLYTTSSQLTLPATQCLAGKSVTCHVKHYTNPSQDVTVPCPVPSTPPTPSPSTPPTPSPSCCHPRLSLHRPALEDLLLGSEANLTCTLTGLRDASGVTFTWTPSSGKSAVQGPPERDLCGCYSVSSVLPGCAEPWNHGKTFTCTAAYPESKTPLTATLSKSGNTFRPEVHLLPPPSEELALNELVTLTCLARGFSPKDVLVRWLQGSQELPREKYLTWASRQEPSQGTTTFAVTSILRVAAEDWKKGDTFSCMVGHEALPLAFTQKTIDRLADWQMPPPYVVLDLPQETLEEETPGANLWPTTITFLTLFLLSLFYSTALTVTSVRGPSGNREGPQY**

VDB33 Light Chain

MGWSCIILFLVATATGVHSSYVLTQPPSVSVAPGKTARITCGGNNIESKSVHWYQQKSRQAPVLVFYDHSDRPSGIPERFSASNSGHTATLIISGVEAGDEADYHCQVWDSDSDHPVFGGGTKLTVLGQPKAAPSVTLFPPSSEELQANKATLVCLISDFYPGAVTVAWKADSSPVKAGVETTTPSKQSNNKYAASSYLSLTPEQWKSHRSYSCQVTHEGSTVEKTVAPTECS**

>VDB11_tmIgG1

MGWSCIILFLVATATGVHSEVQLLESGGGLVQPGGSLRLSCAASGFTFSNYAMTWVRQARGKGLEWVSVMYTGGSSTYYADSVKGRFTISRDNIKNTLYLQMNSLRAEDTAVYYCAKAHCVRGTCNPLEADYWGQGTLVTVSSASTKGPSVFPLAPSSKSTSGGTAALGCLVKDYFPEPVTVSWNSGALTSGVHTFPAVLQSSGLYSLSSVVTVPSSSLGTQTYICNVNHKPSNTKVDKKVEPKSCDKTHTCPPCPAPELLGGPSVFLFPPKPKDTLMISRTPEVTCVVVDVSHEDPEVKFNWYVDGVEVHNAKTKPREEQYNSTYRVVSVLTVLHQDWLNGKEYKCKVSNKALPAPIEKTISKAKGQPREPQVYTLPPSRDELTKNQVSLTCLVKGFYPSDIAVEWESNGQPENNYKTTPPVLDSDGSFFLYSKLTVDKSRWQQGNVFSCSVMHEALHNHYTQKSLSLSPELQLEESCAEAQDGELDGLWTTITIFITLFLLSVCYSATVTFFKVKWIFSSVVDLKQTIIPDYRNMIGQGA**

>VDB11_Light Chain

MGWSCIILFLVATATGVHSQSVLTQPPSASGTPGQRVTISCSGSSSNVGNNHVYWYQQLPGTAPKLLFYGNNQRPSGVPDRFSGSKSGTSASLAISGLRSEDEGDYYCAAWDDSLSGPVFGGGTQLTVLGQPKAAPSVTLFPPSSEELQANKATLVCLISDFYPGAVTVAWKADSSPVKAGVETTTPSKQSNNKYAASSYLSLTPEQWKSHRSYSCQVTHEGSTVEKTVAPTECS**

>CR6261_tmIgG1

MEWSWVFLFFLSVTTGVHSEVQLVESGAEVKKPGSSVKVSCKASGGPFRSYAISWVRQAPGQGPEWMGGIIPIFGTTKYAPKFQGRVTITADDFAGTVYMELSSLRSEDTAMYYCAKHMGYQVRETMDVWGKGTTVTVSSASTKGPSVFPLAPSSKSTSGGTAALGCLVKDYFPEPVTVSWNSGALTSGVHTFPAVLQSSGLYSLSSVVTVPSSSLGTQTYICNVNHKPSNTKVDKKVEPKSCDKTHTCPPCPAPELLGGPSVFLFPPKPKDTLMISRTPEVTCVVVDVSHEDPEVKFNWYVDGVEVHNAKTKPREEQYNSTYRVVSVLTVLHQDWLNGKEYKCKVSNKALPAPIEKTISKAKGQPREPQVYTLPPSRDELTKNQVSLTCLVKGFYPSDIAVEWESNGQPENNYKTTPPVLDSDGSFFLYSKLTVDKSRWQQGNVFSCSVMHEALHNHYTQKSLSLSPELQLEESCAEAQDGELDGLWTTITIFITLFLLSVCYSATVTFFKVKWIFSSVVDLKQTIIPDYRNMIGQGA**

>CR6261_Light Chain

MEWSWVFLFFLSVTTGVHSQSVLTQPPSVSAAPGQKVTISCSGSSSNIGNDYVSWYQQLPGTAPKLLIYDNNKRPSGIPDRFSGSKSGTSATLGITGLQTGDEANYYCATWDRRPTAYVVFGGGTKLTVLGAAAGQPKAAPSVTLFPPSSEELQANKATLVCLISDFYPGAVTVAWKADSSPVKAGVETTTPSKQSNNKYAASSYLSLTPEQWKSHRSYSCQVTHEGSTVEKTVAPTECS**

>MSL109_tmIgG1

MGWSCIILFLVATATGVHSEEQVLESGGGLVKPGGSLRLSCAASGFTFSPYSVFWVRQAPGKGLEWVSSINSDSTYKYYADSVKGRFTISRDNAENSIFLQMNSLRAEDTAVYYCARDRSYYAFSSGSLSDYYYGLDVWGQGTLVTVSSASTKGPSVFPLAPSSKSTSGGTAALGCLVKDYFPEPVTVSWNSGALTSGVHTFPAVLQSSGLYSLSSVVTVPSSSLGTQTYICNVNHKPSNTKVDKKVEPKSCDKTHTCPPCPAPELLGGPSVFLFPPKPKDTLMISRTPEVTCVVVDVSHEDPEVKFNWYVDGVEVHNAKTKPREEQYNSTYRVVSVLTVLHQDWLNGKEYKCKVSNKALPAPIEKTISKAKGQPREPQVYTLPPSRDELTKNQVSLTCLVKGFYPSDIAVEWESNGQPENNYKTTPPVLDSDGSFFLYSKLTVDKSRWQQGNVFSCSVMHEALHNHYTQKSLSLSPELQLEESCAEAQDGELDGLWTTITIFITLFLLSVCYSATVTFFKVKWIFSSVVDLKQTIIPDYRNMIGQGA**

>MSL109_Light Chain

MGWSCIILFLVATATGVHSDIVMTQSPLSLSVTPGEPASISCRSSQSLLHTNGYNYLDWYVQKPGQSPQLLIYLASNRASGVPDRFSGSGSGTDFTLKISRVETEDVGVYYCMQALQIPRTFGQGTKVEIKRTVAAPSVFIFPPSDEQLKSGTASVVCLLNNFYPREAKVQWKVDNALQSGNSQESVTEQDSKDSTYSLSSTLTLSKADYEKHKVYACEVTHQGLSSPVTKSFNRGEC**

>13H11_tmIgG_HC

MGWSCIILFLVATATGVHSQVQLVQSGAEVKKPGASVKVSCKASGYTFTNYYIHWVRQAPGQGLEWMGIIHPSSGGTSYAQKFQGRVTMTRDTSTSTVSMDLSSLRSEDTAVYYCGRAFRILGLSDVFVNDWGQGTVVTVSSASTKGPSVFPLAPSSKSTSGGTAALGCLVKDYFPEPVTVSWNSGALTSGVHTFPAVLQSSGLYSLSSVVTVPSSSLGTQTYICNVNHKPSNTKVDKKVEPKSCDKTHTCPPCPAPELLGGPSVFLFPPKPKDTLMISRTPEVTCVVVDVSHEDPEVKFNWYVDGVEVHNAKTKPREEQYNSTYRVVSVLTVLHQDWLNGKEYKCKVSNKALPAPIEKTISKAKGQPREPQVYTLPPSRDELTKNQVSLTCLVKGFYPSDIAVEWESNGQPENNYKTTPPVLDSDGSFFLYSKLTVDKSRWQQGNVFSCSVMHEALHNHYTQKSLSLSPELQLEESCAEAQDGELDGLWTTITIFITLFLLSVCYSATVTFFKVKWIFSSVVDLKQTIIPDYRNMIGQGA**

>13H11_LC

MGWSCIILFLVATATGVHSDIQMTQSPSSLSASVGDRVTITCRASQGINNYLAWYQQKPGKVPKLLIYAASTLQSGVPSRFSGSGSGTAFTLTILSLQPEDVATYYCQKYNSAPFTFGPGTKVDIKRTVAAPSVFIFPPSDEQLKSGTASVVCLLNNFYPREAKVQWKVDNALQSGNSQESVTEQDSKDSTYSLSSTLTLSKADYEKHKVYACEVTHQGLSSPVTKSFNRGEC**

Kozak: GCCGCCACC

Leader Sequence

Antigen Recognition Sequence

Transmembrane Sequence

>hDC-SIGN

MSDSKEPRLQQLGLLEEEQLRGLGFRQTRGYKSLAGCLGHGPLVLQLLSFTLLAGLLVQVSKVPSSISQEQSRQDAIYQNLTQLKAAVGELSEKSKLQEIYQELTQLKAAVGELPEKSKLQEIYQELTRLKAAVGELPEKSKLQEIYQELTWLKAAVGELPEKSKMQEIYQELTRLKAAVGELPEKSKQQEIYQELTRLKAAVGELPEKSKQQEIYQELTRLKAAVGELPEKSKQQEIYQELTQLKAAVERLCHPCPWEWTFFQGNCYFMSNSQRNWHDSITACKEVGAQLVVIKSAEEQNFLQLQSSRSNRFTWMGLSDLNQEGTWQWVDGSPLLPSFKQYWNRGEPNNVGEEDCAEFSGNGWNDDKCNLAKFWICKKSAASCSRDEEQFLSPAPATPNPPPA

>PDGFRA

MGTSHPAFLVLGCLLTGLSLILCQLSLPSILPNENEKVVQLNSSFSLRCFGESEVSWQYPMSEEESSDVEIRNEENNSGLFVTVLEVSSASAAHTGLYTCYYNHTQTEENELEGRHIYIYVPDPDVAFVPLGMTDYLVIVEDDDSAIIPCRTTDPETPVTLHNSEGVVPASYDSRQGFNGTFTVGPYICEATVKGKKFQTIPFNVYALKATSELDLEMEALKTVYKSGETIVVTCAVFNNEVVDLQWTYPGEVKGKGITMLEEIKVPSIKLVYTLTVPEATVKDSGDYECAARQATREVKEMKKVTISVHEKGFIEIKPTFSQLEAVNLHEVKHFVVEVRAYPPPRISWLKNNLTLIENLTEITTDVEKIQEIRYRSKLKLIRAKEEDSGHYTIVAQNEDAVKSYTFELLTQVPSSILDLVDDHHGSTGGQTVRCTAEGTPLPDIEWMICKDIKKCNNETSWTILANNVSNIITEIHSRDRSTVEGRVTFAKVEETIAVRCLAKNLLGAENRELKLVAPTLRSELTVAAAVLVLLVIVIISLIVLVVIWKQKPRYEIRWRVIESISPDGHEYIYVDPMQLPYDSRWEFPRDGLVLGRVLGSGAFGKVVEGTAYGLSRSQPVMKVAVKMLKPTARSSEKQALMSELKIMTHLGPHLNIVNLLGACTKSGPIYIITEYCFYGDLVNYLHKNRDSFLSHHPEKPKKELDIFGLNPADESTRSYVILSFENNGDYMDMKQADTTQYVPMLERKEVSKYSDIQRSLYDRPASYKKKSMLDSEVKNLLSDDNSEGLTLLDLLSFTYQVARGMEFLASKNCVHRDLAARNVLLAQGKIVKICDFGLARDIMHDSNYVSKGSTFLPVKWMAPESIFDNLYTTLSDVWSYGILLWEIFSLGGTPYPGMMVDSTFYNKIKSGYRMAKPDHATSEVYEIMVKCWNSEPEKRPSFYHLSEIVENLLPGQYKKSYEKIHLDFLKSDHPAVARMRVDSDNAYIGVTYKNEEDKLKDWEGGLDEQRLSADSGYIIPLPDIDPVPEEEDLGKRNRHSSQTSEESAIETGSSSSTFIKREDETIEDIDMMDDIGIDSSDLVEDSFL**
