# Supplementary material for: B cell receptor dependent enhancement of dengue virus infection
Source: PLoS Pathog. 2024 Oct 31;20(10):e1012683. doi: 10.1371/journal.ppat.1012683 (PMC11556684; doi:10.1371/journal.ppat.1012683)
Supplement: Attachment — Submitted filename: ResponseToReviewers.pdf [file ppat.1012683.s015.pdf]

Response to reviewers

1- *Ex vivo secondary heterotypic infection of B cells isolated from volunteers previously infected with DENV 4 did not lead to a substantially higher frequency of DENV+ cells. It is also surprising that monocytes do not become infected, even though myeloid cells are known to be the main viral target in the blood. Can authors explain it? This may be due to the very low MOI used (MOI 0.1) in this important experiment, whereas in Figure 3, an extremely high MOI is chosen. Can the authors explain their choice?*

We thank the reviewer for highlighting this feature of our analysis. We agree with the reviewer that the reason for the low rate of *ex vivo* DENV-1 RVP infection observed in non-B cells in the PBMC samples was the low MOI of the RVP utilized in the assay. This was by design, and was informed by *in vitro* infection experiments – such as those shown in Figure 1 in the manuscript - where we observed the greatest signal:noise ratio in the DENV-1 RVP-based infection assays at an MOI of ~0.1.

s

2- *It is unclear to this reviewer if, in infection assays, binding or infection is being shown, as there is no UV-infection used as a control. Especially for Figure 2, where the infection frequency in B cells is low, it is crucial to show that this is in fact an active infection.*

We emphatically agree with the reviewer that differentiating virion binding/uptake from productive infection is critical for contextualizing the data presented in this manuscript. This is why we elected to utilize a Reporter Virus Particle (RVP) system to quantify DENV infection for the majority of the analysis presented in this study. The expression of the dsRed fluorescent reporter encoded by this RVP will only occur following productive infection of the target cell. We strongly feel that this system offers stringent differentiation of infection from virion binding, as no dsRed protein is contained within the virion and expression of the reporter is completely dependent the replication and translation of the reporter virus genome.

3- *Experiments where BCR is blocked in primary B cells isolated from the DENV-infected cohort would have been crucial to understand the role of BCR in the infection process.*

We completely agree with the reviewer that performing BCR blockade under DENV-infection conditions would be an interesting experiment. However, the limited availability of PBMC from this DENV human-challenge cohort makes this suggested analysis infeasible.

4- *The use of MOI 62 in DENV/Ig/BCR experiments is not justified, and it is unclear how these data are relevant to the in vivo situation.*

We apologize for insufficiently contextualizing the experimental objectives and design process behind the figure highlighted by the reviewer. We felt that it was important to demonstrate DENV infection in primary B cells using "real" DENV as part of this study. However, the low frequency of DENV-specific B cells present in DENV-immune PBMC makes this a challenging experiment utilizing the "direct" infection modality utilized elsewhere in the paper. We utilized a high MOI in this experiment due to the low-probability nature of forming the multi-component/cross-linked immune complex required to attach the DENV virion to the BCR of the primary B cells analyzed in this experiment.

While we feel that this analysis has value, we agree with the reviewer that the assay is quite artificial and does not accurately reflect the "natural" conditions under which DENV-specific B cells could be infected *in vivo*. To this end, we have added significant new data utilizing a DENV-specific and non-DENV-specific B cell lines to demonstrate that human B cell lines expressing DENV-specific BCRs can be directly infected with DENV *ex vivo*. We additionally utilized scRNAseq to quantify the abundance of both positive and negative sense DENV RNA in these cells, demonstrating that this is a productive infection.

However, we have removed the cytokine production data previously shown in Figure 3F of our previous submission as we agree that the relevance of these data to "direct" DENV infection of B cells under more physiologically relevant conditions is unclear.

5- *The purpose of Figure 1G is unclear to this reviewer, 3 points will always yield a high R^2.*

We respectfully disagree with the reviewer on this point. While any 2 points will always yield a high R^2 value, 3 points must be in the same rank-order in both dimensions of a 2-dimentional dataset to achieve a high R^2 value with any correlation analysis. As the point of this figure was to show that the rate of infection achieved via ADE and BDE were in the same rank-order for either IgG or tmIgG constructs, we feel that this figure is both justified and informative.

6- *Based on Figure 2, one could conclude that that none of PBMCs are susceptible to infection as monocytes, which are readily infected in vitro (as many previous studies, some also cited by the authors) are negative here. The reviewer would suggest the use of higher MOI and UV-inactivated virus control.*

As stated above, this assay was intentionally performed with a low MOI to specifically assess the infectibility of DENV-specific B cells and not monocytes. Also, as stated above we strongly believe that the DENV-1 RVP system utilized in our study offer stringent differentiation of infection from virion binding due to the nature of the fluorescent reporter encoded by the reporter virus. Finally, the limited number of PBMC available from this human infection study makes the suggested additional analysis infeasible.

7- *Lines 134-138. To show that BCR that bind DENV could act as a receptor for entry, the authors over-expressed BCR in HEK293 cells. Is there any data on CD79 expression in HEK293 cells? If not, the expressed BCR could merely be binding DENV on the plasma membrane and for it then to move along the membrane to locate clathrin-coated pits for endocytosis. To show that BCR can mediate DENV entry in the same way as Fc gamma receptors, it would be helpful for the authors to consider over-expressing BCR with and without also over-expressing CD79 in HEK293 cells. Alternatively, if HEK293 cells already express CD79, they could consider silencing or knocking out CD79. Without such data, the findings are suggestive but not conclusive of BCR as an entry receptor.*

We wish to thank the reviewer for this comment and suggestion. To further examine and define the contribution of a fully-functional DENV-specific BCR to the process of BCR-mediated infection we have added significant additional analysis of a set of human B cell lines which express either a DENV-specific or ZIKV-specific BCR. These cells were generated in such a fashion that they retain physiological BCR signaling capacity and surface tmIgG. We quantified the infectability of these cells by DENV-2 by both flow cytometry and scRNAseq, demonstrating that the DENV-specific B cell line – but not the ZIKV-specific line – are susceptible in DENV infection. We believe these data – in conjunction with the data already shown in Figure 1 of this manuscript - strongly support the concept that the expression of a DENV-specific BCR renders cells susceptible to DENV infection.

8- *Lines 140-143 and supplemental figure 3. The CMV infection rate even in the control is low in Figure 3C. However, the graph in Figure 3D showed a much larger infection rate than that in Figure 3C. Is there an error in one of the figure panels?*

We thank the reviewer for this question. There was no error in the initial figure panel, but we agree that the format of the dot plot shown in our initial submission was difficult to interpret clearly. We have updated the indicated figure to improve clarity.

9- *Lines 168-187. Were the sets of data described in these paragraphs from experiments using DENV-1 RVP or with DENV itself? If so, was it with DENV-4?*

We apologize for omitting this detail in our initial submission. The analysis described in the section highlighted by the reviewer above was performed with DENV-4 itself, not the DENV-1 RVP. We have updated the manuscript with this information.

10- *Lines 185-187. The authors measured IL8 and TNFa. How were these cytokines chosen over others for analysis? If indeed the authors only measured these two cytokines, then perhaps they could take a more untargeted approach to discover if there are other differences in the host response to infection in B cells compared to other cells, such as monocytes. Please also describe the assay/s used in the methods section.*

We apologize for omitting these details in our initial submission. IL-8 and TNF-a were among a panel of 10 cytokines that were analyzed in this experiment utilizing a multiplex human cytokine panel developed by Mesoscale Diagnostics (MSD). These two cytokines were highlighted due to their previously hypothesized role in dengue pathogeneses. However, we have elected to remove these data from our resubmission, as we agree with the reviewer's previous point that the data are of unclear significance given the route of infection utilized in the assay.

11- *The discussion section is too brief. Perhaps the authors would consider discussing how the affinity of binding of DENV to BCR would have on infection since memory B cells would not have undergone as much affinity maturation as plasma cells. Furthermore, they could also discuss the extent in which B cell response to DENV infection contributes to the pathology of dengue, such as vascular leakage and hemorrhage, or that they function merely as trojan horses for DENV to reach myeloid-derived cells, the response of which drives pathology.*

We apologize for the brevity of the discussion section of the manuscript and agree with the reviewer that these are highly intriguing implications of our model that warrant additional exploration. The Brief Report article format under which this manuscript was submitted has a strict world limit, and we were unable to address these topics to the extent they might deserve in a manuscript format with a larger word limit. These are all topics that warrant additional investigation, are our group is already pursuing studies to address these very questions.

12- *The study does not adequately address whether BCR (like FcR in the context of ADE) leads to (productive) infection. It is unclear whether binding or infection occurs, as there is no UV-infection used as a control in the infection assays and the production of new particles has not yet been assessed. Based on Figure 2, it is difficult to conclude if used PBMCs are infectable at all, as primary monocytes, cells that are readily infected in vitro (many previous studies) are negative here regardless of time of their isolation.*

We strongly believe that the DENV-1 RVP system utilized in our study offers stringent differentiation of infection from virion binding due to the fluorescent reporter encoded by the reporter virus which requires replication and translation of the reporter viral genome for expression. Furthermore, this assay was intentionally performed with a low MOI to specifically assess the infectibility of DENV-specific B cells and not monocytes. However, we have added additional data in Figure 2 of our resubmission, demonstrating that a DENV-specific B cell line contains both negative- and positive-sense DENV RNA after exposure to DENV-2, indicating that the virus is successfully replicating within the permissive cell.

13- *Research has not sufficiently explored whether BCR triggers cytokine production in B cells. It is worth noting that important control conditions for Figure 3C/D, which include DENV + VDB33, DENV + anti-IgG, and VDB3 + anti-IgG, are missing. However, these conditions were featured in the Raji cell experiment, as shown in Figure 3B.*

We agree with the reviewer that the BCR cross-linking system was of insufficiently physiological relevance to reasonably examine the cytokine production profile of DENV-infected B cells. We have therefore removed this data from our revised manuscript.

14- *The introduction and discussion do not elude of any previous research on the processes of direct or antibody-dependent enhancement (ADE) dengue virus (DENV) infection in B cells. One of the studies (referenced by the authors to highlight the importance of B cells) investigated the role of CD300 in primary B cells during direct DENV infection and the inability of these cells to facilitate ADE. However, the text does not address how these findings relate to the authors' hypotheses, or the new results presented.*

We apologize for the brevity of the introduction and discussion section of the manuscript and did not intend to dismiss the importance of the studies highlighted by the reviewer. The Brief Report article format under which this manuscript was submitted has a strict world limit, and we were unable to address these topics to the extent they might deserve in a manuscript format with a larger word limit.

15- *In Figure 2, the authors show that B cells from patients that had been previously infected with DENV3 where more susceptible to infection with DENV1, while their T cells and monocytes were not. It would be a nice*

comparison to see how the monocytes of these individuals reacted to DENV1 infection in the presence of their antibodies to compare BDE and ADE activity from the same individual.

We agree with the reviewer that this is an extremely interesting question. However, the limited number of PBMC available from the dengue human challenge study utilized in this work makes this additional analysis infeasible.

16- *Because it is difficult to isolate DENV-infectable B cells from DENV-immune individuals, the authors developed a BCR-dependent infection assay to study BCR-mediated pathogenesis. Unfortunately, this crosslinking assay is so far removed from the natural condition that it is hard to agree with the authors' assertion that "BCR-dependent infection of B cells results in the production of pro-inflammatory cytokines known to be associated with DENV infection in human patients". Yes, the assay engages BCRs, but these BCRs are from naïve individuals. Perhaps if the assay were better described and discussed, it's inclusion might be more compelling.*

We agree with the reviewer that we overstated the generalizability of the cytokine production data derived from the BCR cross-linking analysis presented in our initial submission. In our revised submission, we have removed the cytokine production data and instead focus on the infection state of the B cells.
