# Supplementary material for: B cell receptor dependent enhancement of dengue virus infection
Source: PLoS Pathog. 2024 Oct 31;20(10):e1012683. doi: 10.1371/journal.ppat.1012683 (PMC11556684; doi:10.1371/journal.ppat.1012683)
Supplement: Attachment — Submitted filename: BDE_response_to_reviewers_11OCT24.pdf [file ppat.1012683.s016.pdf]

<u>Response to reviewers</u>

1. *Line 140. Technically, as the authors only compared the percentage of dsRed positive cells in the study, the data is not dependent on RVP replication but only translation.*

   We agree with the reviewer on this point and have updated our manuscript accordingly (line 141).

2. *Lines 210-212 and Figure 3C,D. The proportion of infected B cells was highest at day 28 compared to both baseline as well as day 90 post-infection. At this early period after convalescence from primary DENV-3 challenge infection, the study participants would likely be refractory to symptomatic secondary DENV infection (Sabin, 1952; Snow et al, Am J Trop Med Hyg 2014). Perhaps this data might suggest that whilst DENV can infect memory B cells via BCR, its role in pathogenesis remains to be determined? Perhaps an inclusion of such a caveat or a more nuanced conclusion would be helpful.*

   We completely agree with the reviewer on this point and apologize for the imprecise language in our prior submission. While the frequency of DENV-specific/DENV-infectible B cell might be highest within the first few weeks/months after a DENV infection, the high titers of neutralizing antibodies in circulation would make the probability of experiencing a secondary infection within this window extremely unlikely. We have revised our submission to highlight this conclusion (line 254).

3. *Line 250. Typographical error at the end of that line.*

   We apologize for the typo and have corrected in our revised submission

4. *The argument that the three data points must be in the same "rank order" to produce a high value reflects a misunderstanding of how the R2 statistic is calculated and interpreted. While rank order may affect the visual representation of data in certain contexts (e.g., when considering monotonic relationships), it is not inherently linked to the calculation of R2 , which measures the proportion of variance in the dependent variable that is explained by the independent variable(s). To clarify, R2 is based on the degree to which the model's predictions align with the observed data, calculated as the ratio of the explained variance to the total variance. This means that R2 is determined by the fit of the model, not by the sequence or rank of the data points. For instance, in the case of a simple linear regression with only three data points, a high R2 can occur if the points are near or on a line, irrespective of whether their values increase or decrease in a specific order. The primary factor is the alignment of the model's predictions with the actual values. Moreover, with such a small sample size, R2 can become an unreliable measure. Overfitting is a significant risk, as even a poorly generalizable model may fit the three points perfectly, resulting in an artificially inflated. This risk exists regardless of the rank order of the points. In conclusion, the notion that the rank order of three points influences R2 misunderstands the statistical principles at play. R2 reflects the fit of the model to the data points, not their sequence, and caution should always be exercised when interpreting R2 values derived from small samples. I therefore advise the authors to remove the R2 analysis or interpret it with more caution.*

   While we agree with the reviewer regarding the specifics of how a R2 statistic is calculated, we still maintain that the analysis has merit as presented in our prior submission. However, the conclusion that we wish to emphasize in the figure – that the rank-order of ADE and BDE activity of our mAbs/tmIgG constructs are the same – does not rely on the correlation analysis. We have retained the figure in our revised submission but have removed the indicated statistical test as requested.
